# Laryngeal Ultrasonography Versus Cuff Leak Test in Predicting Postextubation Stridor

**DOI:** 10.5681/jcvtr.2014.005

**Published:** 2014-03-21

**Authors:** Haleh Mikaeili, Mohammad Yazdchi, Mohammad Kazem Tarzamni, Khalil Ansarin, Maryam Ghasemzadeh

**Affiliations:** ^1^Tuberculosis and Lung Disease Research Center, Tabriz University of Medical Sciences, Tabriz, Iran; ^2^Neurosciences Research Center, Tabriz University of Medical Sciences, Tabriz, Iran; ^3^Department of Radiology, Tabriz University of Medical Sciences, Tabriz, Iran

**Keywords:** Postextubation Stridor, Laryngeal Ultrasonography, Cuff leak Test

## Abstract

***Introduction:*** Although cuff leak test has been proposed as a simple method of predicting the occurrence of postextubation stridor, cut-off point of cuff-leak volume substantially differs between previous studies. In addition, laryngeal ultrasonography including measurement of air column width could predict postextubation stridor. The aim of the present study was to evaluate the value of laryngeal ultrasonography versus cuff leak test in predicting postextubation stridor.

***Methods: *** In a prospective study, all patients intubated for a minimum of 24 h for acute respiratory failure, airway protection and other causes were included. Patients were evaluated for postextubation stridor and need for reintubation after extubation. The cuff leak volume was defined as a difference between expiratory tidal volumes with the cuff inflated and deflated. Laryngeal air column width was defined as the width of air passed through the vocal cords as determined by laryngeal ultrasonography. The air-column width difference was the width difference between balloon-cuff inflation and deflation.

***Results:*** Forty one intubated patients with the mean age of 57.16±20.07 years were included. Postextubation stridor was observed in 4 patients (9.75%). Cuff leak test (cut off point: 249 mL) showed sensitivity and specificity of 75% and 59%, respectively. In addition, laryngeal ultrasonography (cut off point for air column width: 10.95 mm) resulted in sensitivity and specificity of 50% and 54%, respectively. Positive predictive value of both methods were <20%.

***Conclusion:*** Both cuff leak test and laryngeal ultrasonography have low positive predictive value and sensitivity in predicting postextubation stridor and should be used with caution in this regard.

## Introduction

Endotracheal intubation is commonly used for respiratory support in intensive care unit (ICU).^1^ However, intubation/extubation may lead to the development of complications such as postextubation stridor (PES), one of the most frequent causes of reintubation, prolonged mechanical ventilation, and increased morbidity in the ICU patients.^2-9^ The incidence of PES ranges between 6 and 37% in intubated ICU patients, and is up to 22% in patients who are endotracheally intubated for more than 24 hours.^4,10-13^ Factors associated with the development of PES include older age, female gender, size of endotracheal tube, presence of cuffed tube, prolonged intubation period, presence of an underlying airway disease, traumatic intubation, tracheal aspiration, tube mobility and patient fighting against the endotracheal intubation or trying to speak.^4,12-16^

Diagnosis of PES is of significant clinical importance as these patients can benefit from close monitoring and specific therapies. Nonetheless, there is no consensus on a method to identify patients at risk of PES. Cuff leak test (CLT), illustrating a leak around the endotracheal tube with the cuff deflated, has been proposed as a simple method of predicting the occurrence of PES.^4,16-20^ CLT consists of deflating the balloon cuff of the endotracheal tube in order to assess the air leak around the tube, permitting an indirect evaluation of upper airway patency. A reduced cuff-leak volume identifies a population at increased risk for the development of PES. However, cut-off point of the cuff-leak volume substantially differs between previous studies and the controversial results may cause physicians to make difficult decisions regarding extubation if the CLT is positive.^16,20^

There is a substantial need for defining risk of PES development through other methods. Although Ding and colleagues observed that laryngeal ultrasonographic findings including the air column width could predict PES, their study was limited by the small number of patients in the stridor group.^21^ The aim of the present study was to evaluate the value of laryngeal ultrasonography (US) versus CLT in predicting the PES.

## Materials and methods

In this prospective study, all patients admitted to and intubated in the neurology and medical ICUs at Imam Reza Hospital, Tabriz University of Medical Sciences, Tabriz, Iran between February 2009 and February 2010 were included. The inclusion criteria were intubation for a minimum of 24 h for acute respiratory failure, airway protection and other causes. Patients primarily intubated for the upper airway obstruction or vocal cord paralysis with clinical presentation of stridor were excluded. The present study was approved by the institutional review board. Informed consent was obtained from the patients or their relatives.

Patients were evaluated for PES and need for reintubation after extubation. PES was defined as the presence of a high-pitched inspiratory wheeze requiring medical intervention. Associated with respiratory distress within 24 hours of extubation, PES was accompanied with a respiratory rate >30/minute or increase by >10/minute from baseline.^16^ All assessments for stridor and need for reintubation was made by the ICU physicians who were blinded to the previously obtained measurements.

### CLT

The cuff leak was measured when the patient presumed ready for extubation. The procedure was performed according to the protocol proposed by Miller and Cole.^16^ Before the test measurement, oral and endotracheal secretions were suctioned, and the ventilators were placed on the assist-control mode. Tidal volumes were measured with the cuff inflated and deflated. Six subsequent breath cycles were measured and the average value was calculated. The leak volume was defined as a difference between expiratory tidal volumes with the cuff inflated and deflated.

### Laryngeal Ultrasonography (US)

The laryngeal US was performed with a Medisone-accuvix-v10-probe liner (7/5 MHz) for the visualization of the vocal cords according to the protocol described by Ding and colleagues.^21^ All US measurements were performed by an expert radiologist (M.K.T) who was blinded to the results of the CLT. Patients were put in supine position while the neck was hyper-extended. The test was performed with the same settings as the CLT with the balloon cuff inflated and deflated. The laryngeal air column width was defined as the width of air passed through the vocal cords as determined by US. It was recorded for three consecutive times, and the averaged value was recorded. The air-column width difference (ACWD) was the width difference between balloon-cuff inflation and deflation‏.

### Data analysis

Data were presented as median (interquartile range), mean ± standard deviation (SD), or frequency (percentage). Statistical analysis was performed with SPSS 17.0 (SPSS, Chicago, Illinois) using Student’s t, Mann-Whitney U test, chi-square test and Fisher’s exact test, wherever appropriate. Receiver operating characteristic (ROC) curve analysis was conducted to calculate sensitivity, specificity, positive and negative predictive value (PPV and NPV) of CLT and laryngeal US in predicting PES. To better compare our results with those of the similar studies, previously proposed cut off points for CLT (110 mL and 130 mL) were evaluated in predicting PES.^9,17^ A *P* value <0.05 was considered statistically significant.

## Results

In this study, 41 intubated patients (male/female: 26/15) with the mean age of 57.16±20.07 years were included. PES was diagnosed in 4 patients (9.75%). Table 1  demonstrates patients’ characteristics and correlated variables to PES. There was no significant difference regarding gender, age, interval between intubation and admission to ICU, total intubation period, disease severity score, cuff pressure, CLT, air column width before deflation, and ACWD between patients with and without PES (*P*>0.05, Table 1 ).

**Table 1:** Patients’ characteristics and correlated variables to PES

	**With PES** **(n=4)**	**No PES** **(n=37)**	***P***
Male:female	1:3	24:13	NA
Age <60 years	1 (25%)	15 (40.54%)	NA
>48 hours interval between intubation and admission to ICU	0 (0%)	14 (37.83%)	NA
Total intubation period >48 hours	4 (100%)	35 (94.59%)	NA
Disease severity score	31 (11.5)	24 (10.5)	0.66
Cuff pressure (cmH_2_O)	33 (13)	34 (14)	0.34
CLT	152 (301)	329 (275.5)	0.48
Air column width before deflation	12 (0.07)	11.5 (3.45)	0.48
ACWD	0.1 (3.55)	1 (2.65)	0.59

PES, postextubation stridor; CLT, cuff leak test; ACWD, air column width difference; NA, not availabale.

Three patients with PES (75%) had cuff pressure >25 cmH_2_O. ROC curve analysis was used to define the cut off point for CLT and air column width measured by laryngeal US in predicting PES (Table 2). Considering the measured cut off points, sensitivity, specificity, PPV and NPV for each parameter in predicting PES were calculated (Table 3 ). CLT in comparison with laryngeal US resulted in better sensitivity, specificity, PPV and NPV in predicting PES. Moreover, previously proposed cut off points for CLT in the literature (110 mL and 130 mL) yielded sensitivity of 25% and specificity of >80% in predicting PES (Table 3 ).

**Table 2:** Laryngeal US and CLT results in all patients and those with PES, n (%)

		**All** **patients** **(n=41)**	**PES** **(n=4)**
Cuff pressure (cmH_2_O)	≤25	10 (24.39)	1 (25)
	>25	31 (75.6)	3 (75)
CLT for PES	Positive (≤249 mL)	18 (43.9)	3 (75)
	Negative (>249 mL)	23 (56.09)	1 (25)
CLT for extubation failure	Positive (≤314 mL)	21 (51.21)	-
	Negative (>314 mL)	20 (48.78)	-
Air column width before deflation (mm)	<10.95	22 (53.65)	2 (50)
	>10.95	19 (46.34)	2 (50)
ACWD (mL)	<0.85	18 (43.9)	2 (50)
	>0.85	23 (56.09)	2 (50)

**Table 3:** Sensitivity, specificity, NPV and PPV of CLT and laryngeal US in predicting PES, (cut off point)

		Sensitivity	Specificity	PPV	NPV
PES	CLT (249 mL)	75	59	17	96
	CLT (110 mL)	25	84	14	91
	CLT (130 mL)	25	81	13	91
	Air column width (10.95 mm)	50	54	11	91
	ACWD (0.85 mm)	50	57	11	91

## Discussion

PES and upper-airway obstruction are multifactorial in etiology and can occur as a result of laryngotracheal edema, intubation trauma, excessive cuff pressure with mucosal ulceration, and prolonged intubation with secondary inflammation and granuloma formation.^22^ In this prospective study, we evaluated the role of CLT and laryngeal US measuring ACWD in predicting the PES. We observed that both methods had low sensitivity and specificity, although CLT showed higher sensitivity (75%) in predicting PES. Moreover, the low PPV values for both methods might be indicative of their low ability to accurately diagnose PES.

There is still debate on cut off points for CLT in predicting PES. Miller and Cole showed that a cuff leak volume of <110 mL might be indicative of patients at risk for PES.^16^ Jaber and colleagues found increased risk of PES in cuff leak volume values of <130 mL.^4^ Most studies on cuff leak volume documented high specificity and negative predictive values, implying that patients with a cuff leak volume above a certain threshold had low probability of developing PES.^4,16,23^ Nevertheless, low sensitivity and PPV of these methods might be indicative of their limited values in predicting PES. Considering their cut off points (<110 mL and <130 mL), we observed similar findings. However, our results showed that CLT <249 mL, a value much higher than the proposed values in the previous studies, had high sensitivity and NPV, and low PPV. In contrast, Engoren reported that all three PES cases had CLT values of >310 mL.^20^ Altogether, clinical decision-making to start PES treatment merely based on CLT results might be challenging.

There is a need for other methods to predict patients at risk of postextubation complications. Laryngeal US to measure the air column width prior to cuff deflation and ACWD is proposed as a possible method to predict PES and laryngeal edema.^21,24^ Ding and colleagues observed that patients with PES had significantly lower air column width and ACWD compared with non-PES patients.^21^ In contrast, we failed to find significant differences in these parameters between groups. However, we observed that both air column width and ACWD had similar low sensitivity and specificity in predicting PES. Comparing these two methods, it seems that CLT still has better sensitivity and specificity compared with the laryngeal US in predicting PES.

In conclusion, both CLT and laryngeal US might have low sensitivity and PPV in predicting PES and should be used with caution in this regard.

## Acknowledgments

This research was financially supported by Tuberculosis and Lung Disease Research Center, Tabriz University of Medical Sciences, Iran. The authors have no conflicts of interest.

## Ethical issues

All patients gave written informed consents and the study was approved by our local Ethics Committee.

## Competing interests

The authors of the present work declare that there is no conflict of interest.
